# Exploring Healthcare Professionals’ Approaches to Promoting Physical Activity and Reducing Sedentary Behaviour in Clinical Paediatric Populations in South Wales

**DOI:** 10.3390/healthcare14121801

**Published:** 2026-06-22

**Authors:** Amie B. Richards, Rachel L. Knight, Kelly A. Mackintosh, Joanne Hudson, Sarah Denford, Melitta A. McNarry

**Affiliations:** 1 Welsh Institute of Physical Activity, Health and Sport (WIPAHS), Wales, UK; k.mackintosh@swansea.ac.uk (K.A.M.); joanne.hudson@swansea.ac.uk (J.H.); m.mcnarry@swansea.ac.uk (M.A.M.); 2 Applied Sports, Technology, Exercise and Medicine (A-STEM) Research Centre, Swansea University, Swansea SA2 8PP, UK; 3 Ysbyty Gwynedd, Betsi Cadwaladr University Health Board, Penrhosgarnedd, Bangor LL57 2PW, UK; 4 NIHR Health Protection Research Unit in Evaluation and Behavioural Science, University of Bristol, Bristol BS8 1QU, UK; sarah.denford@bristol.ac.uk; 5 Faculty of Health and Life Sciences, School of Psychological Science, University of Bristol, Bristol BS8 1QU, UK

**Keywords:** health promotion, behaviour change, lifestyle interventions, child health

## Abstract

**Highlights:**

**What are the main findings?**

- Time constraints, limited resources, and gaps in knowledge hinder healthcare professionals’ engagement in physical activity conversations.
- While healthcare professionals are well positioned to deliver trusted and timely advice, variability in current practice and ongoing challenges related to confidence, clarity of role, and system-level support hinder meaningful application.

**What are the implications of the main findings?**

- There is a need to standardise physical activity promotion across healthcare settings by developing clear guidelines and toolkits.
- Integrating physical activity promotion into pre-registration and continuing professional development programmes for healthcare professionals could address knowledge gaps and build confidence.

**Abstract:**

**Background/Objectives**: Physical inactivity is a significant public health challenge among children and young people (CYP), particularly within clinical populations. Whilst healthcare professionals (HCPs) are uniquely positioned to implement behaviour change strategies, there is little evidence of implementation in practice. This study examined the practices of, as well as barriers and facilitators experienced by, HCPs in South Wales, United Kingdom (UK), when promoting physical activity (PA) and reducing sedentary behaviour (SED) in clinical paediatric populations, together with their perceptions and proficiencies in delivering this support. **Methods**: A 32-item questionnaire was completed by 41 HCPs, recruited through professional and clinical networks to generate an opportunistic sample from physiotherapists (*n* = 22), doctors (*n* = 7), occupational therapists (*n* = 4), therapy technical instructors (*n* = 2), and others (*n* = 1 each). Quantitative responses were analysed using descriptive statistics; free-text responses underwent qualitative content analysis. **Results**: The findings revealed that 95% of HCPs discussed PA at some or all appointments, with similar figures reported for SED (90%) and exercise (88%). However, only 63% of participants felt PA was adequately addressed within their services. Barriers included time constraints, resource limitations, and knowledge gaps. Key facilitators included training, toolkit availability, and multidisciplinary collaboration. **Conclusions**: This study highlights the need for system-level changes to enable HCPs to deliver consistent, effective messaging that promotes PA and reduces SED, ultimately improving health outcomes for CYP receiving clinical input. Facilitating this approach requires embedding PA promotion into HCP training, expanding referral pathways to CYP, and strengthening multidisciplinary working.

## 1. Introduction

Insufficient physical activity (PA) and excessive sedentary behaviour (SED) are well recognised predictors of numerous negative health outcomes, irrespective of age or underlying health condition(s) [1]. In Wales, United Kingdom (UK), PA levels and engagement in sport and recreational activities are low [2]. Although consistent with global trends [3], this is a pressing health concern, particularly among children and young people (CYP) [4,5], with inactive CYP more likely to become inactive adults [6]. Socioeconomic status, ethnicity, and having a disability, impairment, or long-term illness often exacerbate barriers to equal access to and engagement with community-based PA opportunities, including sport [7]. CYP with disabilities, for instance, are less likely to participate in regular sports or meet PA levels compared to their non-disabled peers, with only 33% engaging in organised sport three or more times per week [5]. Research has shown that children with clinical conditions, such as congenital heart defects [8], respiratory disease, inflammatory/autoimmune conditions, metabolic disorders, cancers [9], and inflammatory bowel disease [10], all engage in lower levels of PA than their peers with no clinical conditions.

Healthcare professionals (HCPs) are uniquely positioned to promote PA amongst target groups as they are trusted sources of health information and regularly see those in need of PA advice [11,12]. Indeed, the healthcare environment has been identified as one of the ‘eight best investments’ for promoting PA [13], with a World Health Organization (WHO) economic analysis (via cost per disability-adjusted life years averted) highlighting PA promotion in primary care as one of the most cost-effective measures [14]. Primary care, and moreover the healthcare system as a whole in the UK, has a wide reach, and within the context of person-centred care, provides opportunities to engage CYP and their families in meaningful discussions about PA. Such conversations could help communicate the need for and benefits and safety of PA, support reductions in SED, and foster long-term health benefits [15]. This aligns with the National Health Service (NHS) “making every contact count” approach, which seeks to integrate brief behaviour change interactions into routine clinical care [16,17,18]. However, whilst there is a potentially effective role for providing brief, structured advice on PA [19], such discussions are often informal, with delivery inconsistent between healthcare providers [20]. Barriers such as a perceived lack of time, insufficient training and knowledge, and a lack of resources and support are often cited as impeding HCPs from engaging in these conversations effectively [21]. HCPs also reported a need for enhanced facilities and equipment and stronger networks with existing services in the local area, such as gyms, to help their endeavours [22]. Similarly, research amongst Swiss paediatric HCPs found that exercise counselling was influenced by clinicians’ knowledge of local PA opportunities, highlighting the importance of awareness and referral pathways for effective PA promotion [23].

It is pertinent to note that while PA promotion is receiving increasing attention in healthcare settings, discussions around SED remain scarcer, particularly within clinical paediatric contexts. This is concerning, given that many CYP with chronic health conditions may spend extended periods engaged in sedentary activities due to fatigue, pain, or parental caution [9,10]. Emerging evidence suggests that high SED levels are linked to adverse outcomes, including poorer cardiometabolic health, reduced physical function, and lower psychological well-being, even among those who meet recommended PA levels [24]. However, HCPs rarely distinguish SED as a separate behavioural target. Importantly, PA, exercise and SED are distinct, albeit related, movement behaviours. Whilst exercise represents a structured form of PA undertaken with the objective of improving fitness or health, SED is not simply the absence of PA and may independently contribute to adverse health outcomes [25]. Failure to recognise these distinctions may result in inconsistent or ineffective health promotion messages being delivered to CYP and their families.

Although barriers to and facilitators of PA promotion have been explored in healthcare settings elsewhere in the UK and internationally, evidence relating specifically to Wales remains limited. This is important given that healthcare policy and service delivery are largely devolved within Wales, creating a distinct context for the implementation of health promotion initiatives. Understanding how HCPs in Wales currently approach PA and SED discussions is, therefore, necessary to inform locally relevant policy, workforce development and service improvement strategies. Little is known about how HCPs in paediatric clinical settings in Wales promote PA, discuss exercise, and address SED within routine clinical care, or whether these distinct movement behaviours are consistently understood and communicated in practice. Therefore, this study seeks to examine current practices, barriers, and facilitators experienced by HCPs in South Wales, United Kingdom (UK), when promoting PA and reducing SED in clinical paediatric populations, as well as their perceptions and proficiencies in delivering this support. Through gaining a deeper understanding of these factors, the study aims to inform strategies for enhancing HCP engagement, improving the quality of patient interactions, and ultimately contributing to better health outcomes for CYP.

## 2. Materials and Methods

### 2.1. Study Design

A cross-sectional survey was employed to capture HCPs’ experiences of promoting PA and reducing SED in clinical paediatric populations. A 32-item questionnaire adapted from the work of Denford et al. (2020) [22], alongside study-specific items designed to explore HCPs’ perspectives, proficiency, and practices relating to PA promotion, exercise promotion, and SED reduction, was employed. Additional items examined topics discussed with patients and families, barriers to and facilitators of PA-related conversations, the provision of exercise programmes, and factors perceived to influence PA participation among paediatric patients. Basic demographic information, including gender, age, profession and work/clinic location, was also collected. The questionnaire underwent review by the research team, comprising researchers with expertise in physical activity, paediatric health, and survey-based research, to assess item relevance, clarity and comprehensiveness. This process was undertaken to establish face and content validity prior to distribution [26]. The final questionnaire is provided as Supplementary File S1.

### 2.2. Data Collection

Ethics approval was granted by Swansea University Research Ethics Committee (approval number: 1 2023 6688 5708). The survey was distributed via the JISC Online Survey Platform (v2). Participants were recruited through professional networks, such as the Welsh Institute of Physical Activity, Health and Sport (WIPAHS) and the Royal College of Paediatrics and Child Health (RCPCH), via social media platforms such as X and LinkedIn and at HCP training workshops. Eligibility criteria consisted of being employed in a healthcare role, being involved in paediatric care, and being fluent in English. Before participation, all respondents provided informed consent electronically.

### 2.3. Data Analysis

Quantitative data, including demographic information and responses to closed and Likert-scale questions, were analysed using descriptive statistics to identify trends. Free-text responses from all eight open-ended questions were analysed using a manifest approach to qualitative content analysis (QCA) [27]. All responses were included in the analysis. For each question, one author (RLK) systematically coded responses at the word or phrase level within Microsoft Excel, focusing on the explicit meaning of the data. Inductively generated codes, which were refined through an iterative process, were then grouped into mutually exclusive categories for each question. A second researcher (ABR) reviewed the coding and categorisation process, with any discrepancies discussed and resolved through consensus. Frequency counts were subsequently conducted to quantify the number of participants reporting each category. This process enhanced analytical credibility and trustworthiness [28].

## 3. Results

The final sample consisted of 41 HCPs from South Wales, UK, representing clinicians from various professions and specialties within the healthcare system. Participants were from a range of age groups and paediatric specialties, including more females (*n* = 35) than males (*n* = 6) and more physiotherapists (*n* = 22) than any other profession (Table 1).

**Figure 1:**
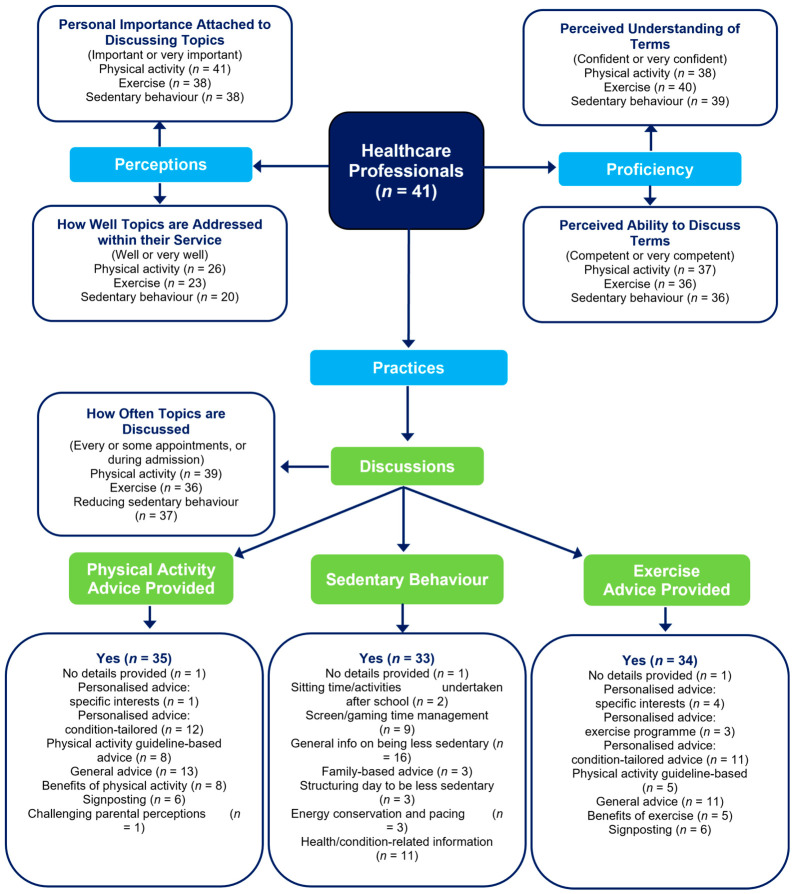
Participant characteristics and results relating to HCPs’ perceptions, proficiency, and practices regarding PA and exercise promotion and SED reduction with CYP.

Findings relating to HCPs’ perceptions, proficiency, and practices regarding PA and exercise promotion and SED reduction with CYP are represented visually (Figure 1). These findings, along with other core areas explored within the survey, are further outlined in the following section. Direct quotations to illustrate the findings from the QCA of the free-text question responses are presented in italics, along with the corresponding participant number.

### 3.1. Perceptions of Importance

The majority of respondents considered discussing PA, exercise, and SED with their patients as highly important. Specifically, 100% of participants (41/41) indicated that discussing PA was ‘important’ or ‘very important’, while 93% (38/41) assigned similar importance to discussing both exercise and SED. Despite this, discrepancies emerged regarding how well HCPs perceived these topics were addressed within their services. Only 63% of participants (26/41) felt PA was adequately discussed, with this figure reducing to 56% (23/41) for exercise and 49% (20/41) for SED, highlighting a notable gap between perceived importance and actual clinical practice.

### 3.2. Proficiency of Understanding and Ability

Respondents’ perceived understanding of the terminology associated with PA, exercise, and SED was high, with 93% (38/41), 98% (40/41), and 95% (39/41), respectively, feeling ‘confident’ or ‘very confident’ in their interpretation of the terms. Likewise, although figures were slightly lower (see Figure 1), the majority of respondents felt ‘competent’ or ‘very competent’ in having discussions about these topics. However, after reviewing definitions, whilst responses remained similar for PA and exercise, confidence and competence in discussing SED dropped to 80% (33/41) and 78% (32/41), respectively.

### 3.3. Discussion Practices

Participants reported varying discussion frequencies of these topics. PA was most frequently mentioned, with 95% of respondents (39/41) discussing it either at every or some appointments. These conversations were, however, often contextual, with one participant noting: *“Depending on how frequently I see [the] patient, I may not discuss [PA] at each appointment but will always discuss with every patient”* (Participant 3, Occupational Therapist). Where it was noted that PA was only discussed in ‘some appointments’, this was attributed to a variety of reasons from the type of clinical condition, to whether the patient was new to their caseload or a long-term patient, *“talk about it more with newer patients,”* (Participant 32, Physiotherapist), appointment frequency, and preferring that such *“conversations [are] elicited by the family”* (Participant 14, Physiotherapist).

Discussion patterns were similar for the topics of exercise and SED, with 88% (36/41) and 90% (37/41), respectively, discussing these at every or some appointments, and always during inpatient admissions. Reasons for not discussing exercise or SED at every appointment mirrored those for PA. For exercise, there were additional considerations cited around conversations needing to be *“relevant to the patient at that time, at that age”* (Participant 39, Physiotherapist) and *“beneficial”* (Participant 14 Physiotherapist) within the wider conversation. For SED, one respondent reported never discussing this with patients and another stated feeling that for this topic, *“conversations are difficult”* (Participant 10, Physiotherapist) and they have to discuss this *“with parents as my patients aren’t cognitively able to understand”* (Participant 9, Therapy Technical Instructor).

Where participants reported providing advice on PA, exercise, and SED, a detailed breakdown of the advice given is presented in Figure 1.

### 3.4. Topics Advised on by HCPs and Barriers to Having Discussions

In total, 85% of participants (35/41) reported giving advice on PA, 78% on exercise (32/41), and 80% on SED (33/41). The most common times when advice on PA or SED was given related to queries about the child’s current PA levels (100%; 35/35) or SED (85%; 28/33), followed by encouragement to be more active and less sedentary (100%; 35/35 and 91%; 30/33, for PA and SED, respectively). For exercise-based discussions, queries about participation (94%; 34/34) and changes the child would like to make (88%; 30/34) were most prevalent. Across all topics, four common barriers were identified during appointments or clinical contact: (i) time and resource limitations; (ii) a lack of knowledge or confidence; (iii) other competing priorities, i.e., immediate medical concerns; and (iv) allowing discussion instigation to be led by the patient/families.

### 3.5. Factors Perceived to Prevent or Encourage Patients’ Physical Activity

HCPs selected a variety of reasons why they perceived that patients were regularly (at least once per week) prevented from being physically active, with the most frequently selected being a lack of enjoyment of activity (63%; 26/41), a preference for other activities (66%; 27/41), and concerns about exacerbating symptoms (63%; 26/41). Free-text responses included *“finances”* (Participant 3, Occupational Therapist), *“lack of provision for those with disabilities”* (Participant 4, Doctor) and *“family challenges with other children in family, working pressures of family and parental health”* (Participant 11, Physiotherapist). Conversely, the factors HCPs perceived to be the most likely to increase engagement were peer support and opportunities that allow engagement alongside similar-aged CYP or those with shared conditions (90%; 37/41), enjoyable options being available (78%; 32/41), the involvement of family in the activity (78%; 32/41), and educating families about the safety (61%; 25/41), benefits (66%; 27/41) and most appropriate intensity (61%; 25/41) of PA.

### 3.6. Barriers to Offering Prescribed Exercise Programmes

Prescribed exercise programmes were reported to be offered within only 56% (23/41) of the clinical areas represented. Among the 44% who did not offer such programmes, time and resource limitations (35%; 8/18) and preferring to focus on PA and/or general function (28%; 5/18) were presented as key reasons for exercise-programme prescription not occurring: *“Lack of space and resource… lack of staff members to run prescribed programmes”* (Participant 27, Physiotherapist). Additional space/facilities (32%; 13/41), resources (staff/funding/time; 32%; 13/41), and support from/opportunities within the community (24%; 10/41) were the most commonly identified themes from free-text responses regarding factors that would facilitate provision within their service.

Resolving resource constraints was identified as a step to improve HCPs’ ability to facilitate PA and/or exercise at a patient’s home, with greater knowledge/training (27%; 11/41) also deemed important. While one participant referred to the value of being able to visit families at home, the majority highlighted a need for practical resources to support home-based engagement, such as information leaflets, accessible online videos, greater awareness of inclusive local opportunities, and additional staffing:

*“Clear, fun, interactive resources to teach children and families about recommended types/duration of physical activity for each age group. More information of groups available e.g., summer camps, youth groups, play groups (and information to be gathered in a single place for ease of reference)”*

(Participant 4, Doctor)

Along with increased knowledge, to promote SED reductions and PA increases both at their facility and within patients’ homes, clinicians would also value having access to improved information/structured toolkits (29%; 12/41 and 24%; 10/41, respectively) to facilitate engagement.

## 4. Discussion

This study provided critical insights into the practices, barriers, and facilitators HCPs face when promoting PA and reducing SED among clinical paediatric populations in South Wales, UK. Despite strong recognition of the importance of these topics, the findings highlight variation in practice and important systemic and individual barriers to PA promotion in healthcare settings [19,20].

Our findings revealed that after respondents reviewed the definition of SED, confidence and competence in discussing this topic decreased. SED is a relatively novel concept in public health discourse compared to PA and exercise. Importantly, this finding suggests that HCPs may overestimate their understanding of SED until presented with a formal definition. Whilst PA and exercise are relatively well-established concepts within healthcare, SED is often incorrectly viewed simply as the absence of exercise rather than a distinct movement behaviour with independent health implications. This distinction is particularly important given growing evidence that prolonged SED is associated with adverse physical and mental health outcomes in children and young people, irrespective of participation in moderate-to-vigorous PA [29]. Consequently, improving HCPs’ understanding of SED may represent an important, yet under-recognised, target for professional development and training.

A lack of understanding around the nuances between different terminology and concepts could have implications for patient engagement. Netherway et al. (2021) argued that inconsistent messaging can confuse patients and undermine their engagement with PA recommendations [21]. Standardised definitions and training are, therefore, essential to ensure HCPs deliver clear, actionable advice. However, what patients interpret as ‘physical activity’ may differ from clinical or public health definitions, often shaped by the commercialised fitness industry’s emphasis on structured exercise or high-intensity workouts [30]. This can result in a narrow understanding that excludes everyday activities such as walking, household chores or active play, which are particularly relevant for clinical paediatric populations and those with chronic conditions [22]. To address this, HCPs not only need to use accurate and inclusive language but also explain what PA entails in accessible and personalised terms.

In addition to training and education on appropriate terminology use, the present findings suggest that conceptual understanding of PA, exercise, and SED may itself represent a barrier to effective health promotion practice. Although many participants reported feeling confident in their general understanding of PA, their responses revealed sizable challenges in applying this knowledge within clinical practice. This echoes the findings of Netherway et al. (2021), who noted that pre-registration training for HCPs often fails to adequately address PA promotion [21]. Strengthening this component of professional education, as advocated by Lobelo et al. (2020), may help to close these knowledge-to-practice gaps and increase practitioner confidence in delivering effective PA advice [19].

The barriers identified in this study, namely time constraints, limited resources, and gaps in knowledge, align closely with findings from previous research conducted both in the UK [17,21,31] and internationally [32,33]. Netherway et al. (2021) highlighted that insufficient training and low confidence continue to hinder HCPs’ engagement in PA conversations, despite widespread recognition of their importance [21]. Similarly, Lowe et al. (2018) reported that brief PA behaviour change approaches are often poorly understood, with many HCPs unsure of how to incorporate them effectively into routine practice [31]. In our study, 35% of HCPs cited time and resource limitations as key barriers, echoing findings by Lobelo et al. (2020), who emphasised that the availability of resources substantially influences the likelihood of PA discussions occurring in clinical contexts [19]. Additionally, several participants described a tendency to wait for patients or families to initiate these conversations, an issue commonly observed in the literature, where HCPs often take a reactive rather than proactive approach when competing clinical priorities are at play [21,34]. Waiting for patients to initiate the conversation might, in part, be explained by practitioners’ belief that a major barrier to patients undertaking PA is their own motivation [35]. HCPs within this study selected various perceived barriers to patients’ PA, including a preference for other activities, followed by a lack of enjoyment and concerns about exacerbating symptoms. As highlighted in the recent ‘We Are Undefeatable’ report, it is important to recognise the clear discrepancy between barriers that patients face and barriers that practitioners perceive patients face, making the need to increase knowledge and understanding of health behaviour changes at a practitioner level vital.

### 4.1. Strengths, Limitations and Future Recommendations

This study focuses on a specific geographic context, which is a key strength, providing valuable insight into the practices and challenges faced by HCPs in South Wales, UK. However, the descriptive nature of the study, relatively small sample size, and regional focus limit the generalisability of the findings. The voluntary recruitment strategy may have introduced selection bias, potentially attracting HCPs with a pre-existing interest in PA promotion. In addition, physiotherapists were overrepresented within the sample, which may have influenced the findings and limited representation of other professional groups. Also noteworthy is that the survey was self-report, which increases the potential for responder bias within the findings, and no objective measures of clinical practice were included. This likely masks a lack of depth in HCPs’ understanding of PA, exercise, and SED, and, importantly, their differences, which may underpin the discrepancies observed in the present study between perceived competence and actual delivery of advice. Furthermore, whilst HCPs reported the frequency with which PA was discussed with patients, the study was unable to assess the quality, appropriateness, or effectiveness of these conversations. Future studies should aim to include larger, more diverse samples to capture a broader range of perspectives. Future research should examine the effectiveness of different communication strategies in engaging families and addressing barriers to PA, together with exploration of the content of the discussions.

### 4.2. Implications for Practice and Policy

The findings of this exploratory study have several potential implications for practice and policy. Firstly, participants highlighting the variation in practice indicates that there is a need to standardise and enhance PA promotion across healthcare settings by developing clear guidelines and toolkits. Secondly, the findings suggest that integrating PA promotion into pre-registration and continuing professional development programmes for HCPs could address knowledge gaps and build confidence. Thirdly, participants identified challenges relating to signposting and onward referral options, suggesting that expanding community-based initiatives to include age-appropriate options for CYP could enhance the reach and impact of PA promotion efforts. Additionally, greater clarity is needed regarding the specific role of HCPs in promoting PA, particularly relating to the boundaries between general advice, signposting, and when input from specialist exercise professionals would be more appropriate. Clearly defining what is feasible, safe, and appropriate within routine practice may help support HCPs to confidently deliver effective advice without questioning their capability or compromising patient trust. Establishing clear expectations could also help avoid delivery variability and ensure PA promotion is both consistent and well-received by CYP and their families. Collectively, these findings suggest that training, structured toolkits, and clearer referral pathways may yield improvements in PA promotion within paediatric services.

## 5. Conclusions

In conclusion, this exploratory study highlights the central role of HCPs in supporting PA promotion among CYP within routine healthcare encounters. While HCPs are well positioned to deliver trusted and timely advice, the participants self-reported variability in current practice and ongoing challenges related to confidence, clarity of role, and system-level support. Although based on a small sample of HCPs in South Wales, these findings suggest that addressing these issues requires not only improved training and resources, but also clearer integration of PA promotion within existing models of person-centred care. Strengthening this alignment has the potential to enhance the consistency and effectiveness of PA promotion for CYP and their families across healthcare settings.

## Figures and Tables

**Table 1:** Participant characteristics.

Characteristic	Category	n
Gender	Female	35
	Male	6
Age Group (years)	25–29	1
	30–34	9
	35–39	4
	40–44	10
	45–49	7
	50–54	5
	55–60	5
Profession	Physiotherapist	22
	Doctor	7
	Occupational Therapist	4
	Therapy Technical Instructor	2
	Nurse	1
	Dietician	1
	Pharmacist	1
	Clinical Psychologist	1
	Podiatrist	1
	Therapy Assistant Practitioner	1
Paediatric Specialty	Weight Management	7
	General Paediatrics	5
	Rheumatology and/or Chronic Pain	5
	Community	3
	Musculoskeletal	2
	Diabetes and/or Endocrinology	2
	Orthopaedics	2
	Anaesthetics	1
	Cystic Fibrosis	1
	Chronic Illness	1
	General Practice	1
	General Paediatrics and Rheumatology	1
	Metabolic Bone Disease	1
	Oncology and Haematology	1
	Podiatry	1
	Not stated	6

## Data Availability

The data presented in this study are available on request from the corresponding author due to ethical restrictions.

## Supplementary Materials

The following supporting information can be downloaded at: https://www.mdpi.com/article/10.3390/healthcare14121801/s1, Supplementary File S1: Survey.

## Abbreviations

The following abbreviations are used in this manuscript:

PA	Physical Activity
SED	Sedentary Behaviour
UK	United Kingdom
CYP	Children and Young People
HCP	Healthcare Professional
NHS	National Health Service
QCA	Qualitative Content Analysis
