# Supplementary material for: Exploring Healthcare Professionals’ Approaches to Promoting Physical Activity and Reducing Sedentary Behaviour in Clinical Paediatric Populations in South Wales
Source: Healthcare (Basel). 2026 Jun 22;14(12):1801. doi: 10.3390/healthcare14121801 (PMC13299425; doi:10.3390/healthcare14121801)
Supplement: Supplementary file 1 [file healthcare-14-01801-s001.zip › healthcare-4309486-supplementary.pdf]

Supplementary File 1: Survey

a) About you

1. **Gender**

Male	[ ]
Transgender male	[ ]
Female	[ ]
Transgender female	[ ]
Non-Binary	[ ]
Prefer not to say	[ ]

2. **Age** _____________

b) The clinic

1. Locality of clinic: ______________________________________
2. Speciality of clinic: _____________________________________
3. Your position (please circle): Doctor / Physiotherapist / Occupational therapist/ other (please specify): ______________________________________
4. How many children and adolescents are registered in your clinic? ___________________

c) Physical activity, exercise, and sedentary behaviour

1. Using a scale of 1 (not very confident) to 5 (very confident), indicate how confident you are in your understanding of each of the following terms (please circle).

Physical activity

1	2	3	4	5
(not very confident)		(neutral)		(very confident)

Exercise

1	2	3	4	5
(not very confident)		(neutral)		(very confident)

Sedentary behaviour

1	2	3	4	5
(not very confident)		(neutral)		(very confident)

2. Using a scale of 1 (not very competent) to 5 (very competent), indicate how competent you feel to discuss each of the following with your patients (please circle).

Physical activity

1	2	3	4	5
(not very confident)		(neutral)		(very confident)

Exercise

1	2	3	4	5
(not very confident)		(neutral)		(very confident)

Reducing sedentary behaviour

1	2	3	4	5
(not very confident)		(neutral)		(very confident)

Please review the following definitions and consider these when answering the questions that follow:

Physical activity: "Any bodily movement produced by skeletal muscles that results in energy expenditure". (Caspersen et al., 1985).

Exercise: "Physical activity that is planned, structured, repetitive, and purposive in the sense that improvement or maintenance of one or more components of physical fitness is an objective". (Caspersen et al., 1985)

Sedentary Behaviour: "Any waking behavior characterized by an energy expenditure ≤1.5 metabolic equivalents (METs), while in a sitting, reclining or lying posture". (Tremblay et al., 2017)

3. Using a scale of 1 (not very confident) to 5 (very confident), indicate how confident you are in your understanding of each of the following terms (please circle).

   Physical activity

1	2	3	4	5
(not very confident)		(neutral)		(very confident)

   Exercise

1	2	3	4	5
(not very confident)		(neutral)		(very confident)

   Sedentary behaviour

1	2	3	4	5
(not very confident)		(neutral)		(very confident)

4. Using a scale of 1 (not very competent) to 5 (very competent), indicate how competent you feel to discuss each of the following with your patients (please circle).

   Physical activity

1	2	3	4	5
(not very confident)		(neutral)		(very confident)

   Exercise

1	2	3	4	5
(not very confident)		(neutral)		(very confident)

   Reducing sedentary behaviour

1	2	3	4	5
(not very confident)		(neutral)		(very confident)

5. Using a scale of 1 (not important at all) to 5 (very important), indicate the importance that you personally attach to each of the following topics in the healthcare of the patient (please circle).

   Physical activity

1	2	3	4	5
(not important at all)		(neutral)		(very important)

   Exercise

1	2	3	4	5
(not important at all)		(neutral)		(very important)

   Reducing sedentary behaviour

1	2	3	4	5
(not important at all)		(neutral)		(very important)

6. Using a scale of 1 (not at all) to 5 (very well), how well do you feel each of the following topics are addressed within your service (please circle)?

Physical activity

1	2	3	4	5
(not at all)		(neutral)		(very well)

Exercise

1	2	3	4	5
(not at all)		(neutral)		(very well)

Reducing sedentary behaviour

1	2	3	4	5
(not at all)		(neutral)		(very well)

7. If at all, how often do you discuss the following with your patients (please tick as many as appropriate)?

Physical activity

Never [ ]
Once a year [ ]
Only if mentioned by the patient [ ]
At every appointment [ ]
At some appointments [ ]
During inpatient admission [ ]
Other [ ] please specify_______________________________________
If more than one box ticked, please explain:____

Exercise

Never [ ]
Once a year [ ]
When a patient reports exercise difficulties [ ]
Only if mentioned by the patient [ ]
At every appointment [ ]
At some appointments [ ]
During inpatient admission [ ]
Other [ ] please specify________________________________
If more than one box ticked, please explain:____

Reducing sedentary behaviour

Never [ ]
Once a year [ ]
When a patient reports exercise difficulties [ ]
Only if mentioned by the patient [ ]
At every appointment [ ]
At some appointments [ ]
During inpatient admission [ ]
Other [ ] please specify________________________________
If more than one box ticked, please explain: ____

---

8. In the clinic, whose responsibility is it to discuss each of the following topics with the patient (please tick as many as appropriate)?

Physical activity

Doctor	[ ]	Therapy assistant	[ ]
Nurse	[ ]	Health care support worker	[ ]
Physiotherapist	[ ]	Exercise professional	[ ]
Don't know	[ ]		

Other personal please specify: ___________________________________

Exercise

Doctor	[ ]	Therapy assistant	[ ]
Nurse	[ ]	Health care support worker	[ ]
Physiotherapist	[ ]	Exercise professional	[ ]
Don't know	[ ]		

Other personal please specify: ___________________________________

Reducing sedentary behaviour

Doctor	[ ]	Therapy assistant	[ ]
Nurse	[ ]	Health care support worker	[ ]
Physiotherapist	[ ]	Exercise professional	[ ]
Don't know	[ ]		

Other personal please specify: ___________________________________

9. Is advice given to patients on the topic of physical activity?

Yes [ ]
No [ ]

10. If yes, what advice is given
If no, please explain why not____

11. If yes, which of these specific topics are discussed (tick all that apply)

Queries about the child's current activity levels ☐
Queries about any changes in the child's current activity levels ☐
General encouragement for the child to be physically active ☐
Information about how much physical activity the child should do ☐
Types of physical activity the child could do ☐
Other ☐ (please specify) ____

12. Is advice given to patients on the topic of exercise?

Yes [ ]
No [ ]

13. If yes, what advice is given
If no, please explain why not____

14. If yes, which of these specific topics are discussed (tick all that apply)

Queries about the child's current exercise participation ☐
Queries about any changes in the child's exercise participation ☐
Queries about changes the child would like to make or have made ☐
General encouragement for the child to participate in regular exercise ☐
Information about how much exercise the child should do ☐
Types of exercise/sporting activities the child could do ☐
Other ☐ (please specify) ____

15. Is advice given to patients on the topic of sedentary behaviour?

Yes [ ]
No [ ]

16. If yes, what advice is given
If no, please explain why not ____

17. If yes, which of these specific topics are discussed

Queries about the child's current sedentary time ☐
Queries about any changes in the child's sedentary time ☐
General encouragement for the child to be less sedentary and more active ☐
Queries about child's current screen time ☐
General encouragement for the child to have less screen time and be more active ☐
Other ☐ (please specify)____

18. Does the clinic offer a prescribed exercise training programme for patients (please tick)?

Don't know [ ]
Yes [ ]
No [ ]

19. If yes, please describe ____
20. If no, please explain why (e.g., timing, resources, finances, lack of need etc ____________________
21. What would improve your ability to facilitate exercise to be undertaken within the facility by patients? ____
22. What would improve your ability to promote and facilitate exercise and/or physical activity for your patients at home? ____
23. What would improve your ability to promote reductions in sedentary behaviour within the facility for your patients? ____
24. What would improve your ability to promote reductions in sedentary behaviour for your patients at home? ____

25. What do you perceive to be the main reasons your patients are regularly (e.g., at least once a week) **prevented** from being physically active (through exercise or physical activity)

Lack of time ☐
Lack of enjoyment of activity ☐
Tiredness ☐
Unwell ☐
School/homework pressure ☐
Peer pressure ☐
Would rather do something else with spare time ☐

Concerns about appearance ☐
Concerns about exacerbating symptoms ☐
Concerns about the safety of physical activity with their condition ☐
Unclear what type / intensity of physical activity to do ☐
Family concerns ☐
Other (please state)____

26. What do you perceive to be the main factors that would **encourage** your patients (e.g., at least once a week) to be more physically active (through exercise or physical activity)

More time ☐
Enjoyment of activity ☐
Less School/homework pressure ☐
Peer support ☐
Provision of opportunities by your clinic ☐
Provision of opportunities with other children with the same or similar conditions ☐
Provision of information about managing symptoms during physical activity ☐
Education about the safety of physical activity with their condition ☐
Education about the benefits of physical activity with their condition ☐
Education about what type/ intensity of physical activity to do ☐
Family involvement ☐
Other (please state)____
